# *In vitro* culture of red-rumped agouti preantral follicles enclosed in fresh and vitrified ovarian tissues using TCM199 plus different pFSH concentrations

**DOI:** 10.1590/1984-3143-AR2022-0113

**Published:** 2023-06-05

**Authors:** Erica Camila Gurgel Praxedes, Luana Grasiele Pereira Bezerra, Náyra Rachel Nascimento Luz, Andréia Maria da Silva, Alexsandra Fernandes Pereira, Alexandre Rodrigues Silva

**Affiliations:** 1 Laboratório de Conservação de Germoplasma Animal, Universidade Federal Rural do Semi-Árido, Mossoró, RN, Brasil; 2 Laboratório de Biotecnologia Animal, Universidade Federal Rural do Semi-Árido, Mossoró, RN, Brasil

**Keywords:** wildlife, female germplasm, follicular development, cryopreservation, biobank

## Abstract

Considering the relevance of establishing biodiversity conservation tools, the study aimed to investigate the TCM199 supplemented with different follicle-stimulating hormone (FSH) concentrations on survival and development of fresh and vitrified preantral follicles enclosed in red-rumped agouti ovarian tissues cultured *in vitro*. In the first experiment, six pairs of ovaries were fragmented and cultured for 6 days according to groups: 10 ng/mL pFSH (FSH10 group) and 50 ng/mL (FSH50 group). Non-cultured tissues were considered as a control. In the second experiment, vitrified/warmed fragments of four pairs of ovaries were cultured with the best concentration of FSH established (cryopreserved and cultured group). Non-cryopreserved (fresh control group) and cryopreserved but non-cultured (non-cultured group) tissues were used as controls. For both experiments, preantral follicles were evaluated for survival and development using morphological and viability analysis by trypan blue staining. After culturing fresh samples, FSH50 showed a higher percentage of morphologically normal follicles when compared to FSH10 (P < 0.05). This same response was observed for primordial follicles. Regardless of the concentrations of FSH used during *in vitro* culture, no difference was observed regarding the percentage of viable follicles and diameters (P > 0.05). Thus, the FSH50 group was used for second experiment, in which 76.2 ± 7.2% normal preantral follicles previously vitrified was found after 6-day culture, also presenting the highest values (P < 0.05) for morphology of primordial follicles (95.2 ± 4.7%). Nevertheless, *in vitro* culture did not affect the viability and diameter of preantral follicles of cryopreserved tissues (P > 0.05). In conclusion, TCM199 supplemented with 50 ng/mL FSH was efficient in maintaining the *in vitro* survival of fresh and vitrified red-rumped agouti preantral follicles. This was the first study related to the *in vitro* culture of ovarian preantral follicles in this species, aiming to contribute to its conservation.

## Introduction

Given the emergence of the sixth phase of mass extinction (Lueders and Allen, 2020), efforts directed towards the development of biodiversity conservation strategies become a growing demand that involves different sectors of society. Into an ecosystem, the loss of a unique component would represent a great damage for all the species that inhabit there. As a pray for carnivores and a seed disperser, the agouti largely contributes for the equilibrium of their habitats (Hosken and Silveira, 2001). From the 13 catalogued agouti species, only three remain presenting a stable population, including the *Dasyprocta leporina*, the red-rumped agouti, that inhabits Brazilian Caatinga (Emmons and Reid, 2016). At this sense, various efforts related to *ex situ* conservation strategies, as the biobank formation (Castelo et al., 2015; Praxedes et al., 2020; Rodrigues et al., 2021), have been conducted to help on the efforts to maintain the stability of free-living *D. leporina* populations and provide technologies for its captive sustainable breeding (Lall et al., 2020).

Recently, the establishment of a solid-surface vitrification (SSV) protocol for red-rumped agouti ovarian tissue preservation was demonstrated, denoting the possibility of conserving valuable female germplasm in biobanks (Praxedes et al., 2020), especially at the use of an ovarian tissue cryosystem device (Praxedes et al., 2021). Despite the goal reached by these studies, the posterior use of the samples after warming remains a great challenge. At this sense, the development of *in vitro* culture (IVC) systems that provide appropriate conditions for the ovarian preantral follicles (PAFs) to grow (Cecconi et al., 2004) is a step extremely important for the wildlife conservation puzzle.

Due to its rich composition, the Tissue Culture Media 199 (TCM199) has been indicated as an adequate media for ovarian tissue IVC in domestic and wild species (Madboly et al., 2017; Lima et al., 2018). Among the supplements incorporated to the media, the follicle-stimulating hormone (FSH) highlighted for presenting indirect action on follicles initial development through the stimuli of paracrine factors from the ovarian stroma and from the follicles, thus promoting initial development through the stimulation of cell proliferation and steroid synthesis (Martins et al., 2008). Because the heterogeneity of the wild species physiology, however, the determination of adequate concentrations of media supplements that support the development of ovarian follicles is a key point for the establishment of effective IVC systems. In fact, the FSH concentration in the medium varies even for domestic species, which can present distinct responses. Matos et al. (2007) reported the use of 50 ng/mL FSH in culture media in goat PAFs grow, while Ferreira et al. (2020) using concentrations varying from 0 to 100 ng/mL observed that goat PAFs did not improve the overall outcome. Additionally, there are different sources of FSH used in *in vitro* culture of PAFs, such as pituitary FSH (pFSH) and recombinant human FSH [rFSH – Magalhães et al. (2009a, b)]. Nevertheless, in the few studies that compare both sources under the same conditions, no difference was observed for the use of pFSH and rFSH during the *in vitro* culture of PAFs (Ferreira et al., 2020).

Therefore, the aim was to evaluate the effect of an IVC system based on the use of TCM199 supplemented with different concentrations of FSH (10 and 50 ng/mL) on the follicle survival and development of red-rumped agouti PAFs enclosed in ovarian tissues, using morphological and viability analysis. Then, the effect of this IVC system was checked on the same parameters of red-rumped agouti PAFs subjected to vitrification.

## Methods

### Ethical considerations

The Ethics Committee of Federal Rural University of Semi-Arid (UFERSA, no. 23091.005916/2015-74) and the Chico Mendes Institute for Biodiversity Conservation (no. 66618-1) approved the experimental protocols. Unless stated otherwise, chemicals and media were purchased from Sigma Chemical Co. (St. Louis, MO, USA).

### Animals and collection of ovaries

Ten mature red-rumped agouti females aging 2–3 years and weighing 2.2–2.7 kg, from the Center of Multiplication of Wild Animals, UFERSA (Mossoró, Brazil; 5º10'S, 3º10 ' W), were used for the study. These individuals were distributed as six females for the first experiment, and four for the second one. For ovarian collection, animals were fasted for 12 h, restrained using a hand net, and premedicated with intramuscular administration of 15 mg/kg ketamine hydrochloride (Ketalar; Pfizer, São Paulo, Brazil) and 1 mg/kg xylazine hydrochloride (Rompun; Bayer, São Paulo, Brazil). After 15 min, anesthesia was induced with intravenous administration of sodium thiopental (Thiopentax; Cristalia, São Paulo, SP, Brazil), and the animals were subsequently euthanized with intravenous 1 mL/kg potassium chloride (Castelo et al., 2015). Immediately thereafter, there was opening of the abdomen and recovery of the ovaries that were washed in 70% ethanol, followed by two lavages in Minimum Essential Medium [MEM, Gibco-BRL, CA, USA – Praxedes et al. (2020)]. The ovaries were transported within 1 h to the laboratory in MEM at 4 °C.

### Experimental design 1

**Figure 1:**
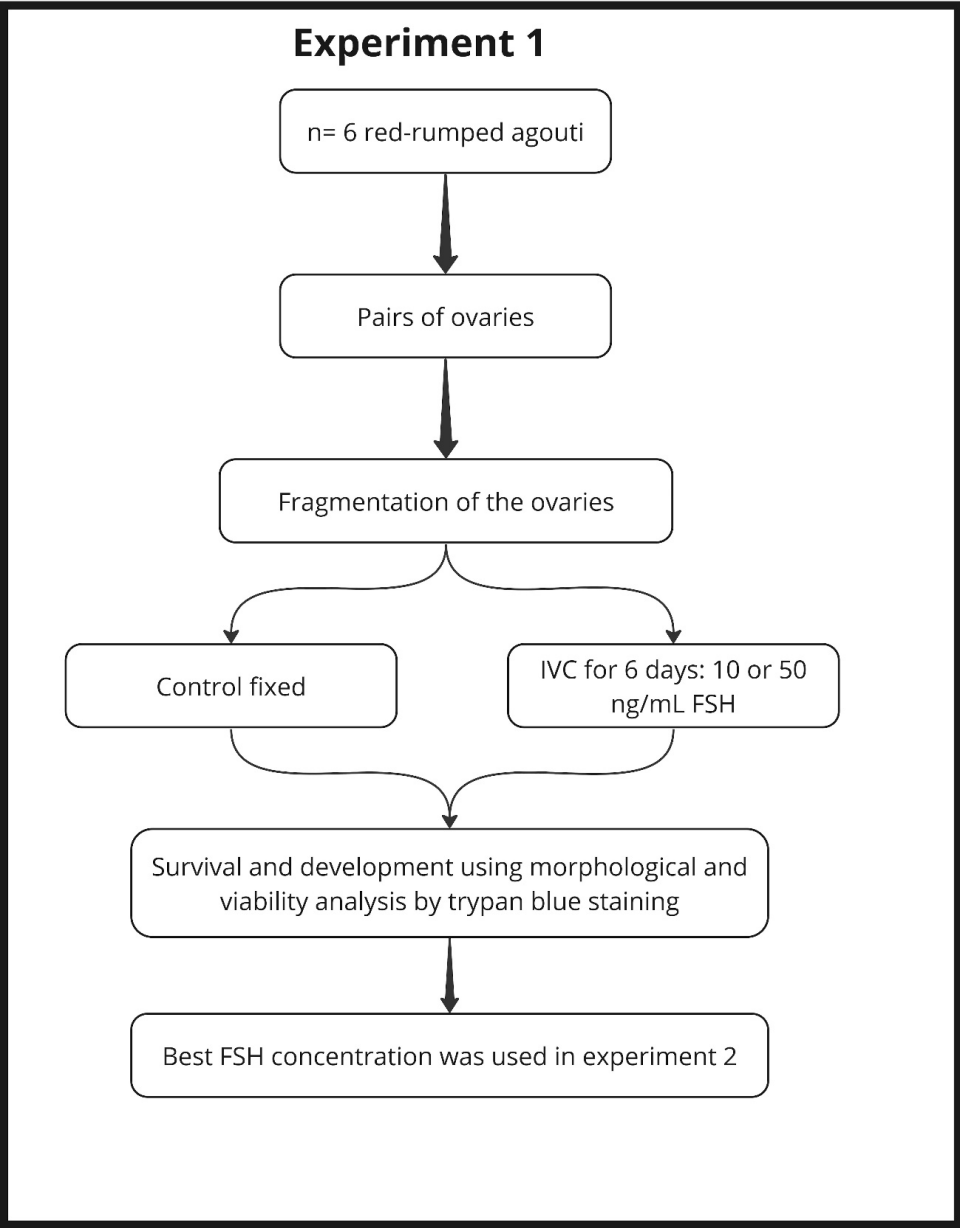
Experimental design (first experiment) to assess the effect of different pFSH concentrations on the morphology and viability of red-rumped agouti preantral follicles after *in vitro* culture for six days.

**Figure 2:**
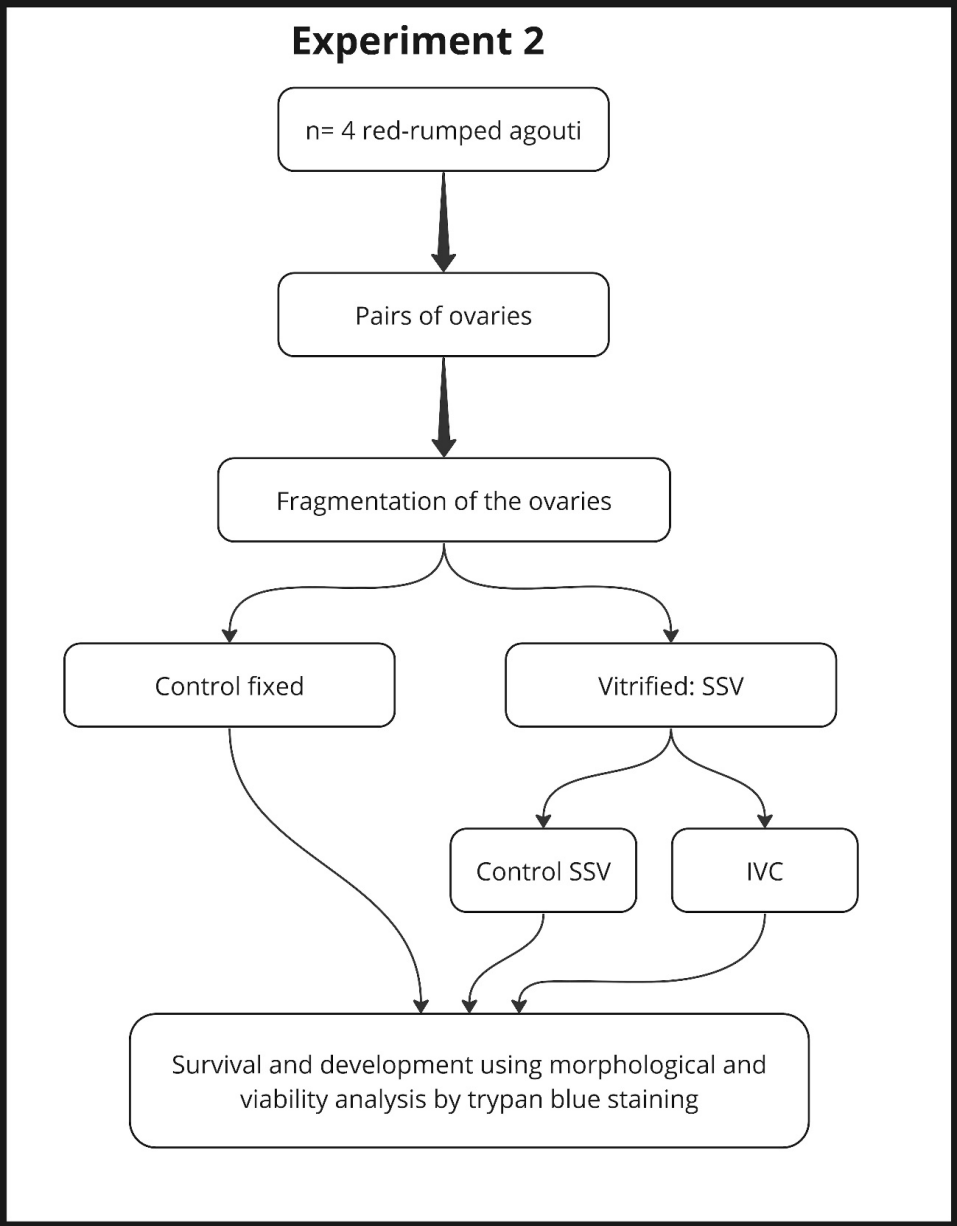
Experimental design (second experiment) to evaluate the effect of solid-surface vitrification (SSV) on red-rumped agouti preantral follicles morphology, development and viability following *in vitro* culture for six days.

Two experiments were designed (Figure 1 and Figure 2). In the first experiment, pairs of ovaries were fragmented and cultured *in vitro* for 6 days according to the groups: 10 ng/mL pFSH (FSH10 group) and 50 ng/mL (FSH50 group). Non-cultured tissues were considered as a control group. The PAFs were evaluated for survival and development using morphological and viability analysis (Figure 1).

#### *In vitro* culture of ovarian fragments – experiment 1

Initially, ovarian tissues of each animal were divided in 12 fragments (9.0 mm^3^ = 3 × 3 × 1 mm). Four fragments constituted the fresh control group that was immediately analyzed, and the others were distributed for cultured groups (4 fragments per group). Subsequently, the four fragments were used in the evaluations, being two fragments used for morphological evaluations and the other two fragments used for viability evaluations. For the IVC, fragments were allocated in plates placed in 24-well culture dishes containing 1.0 mL of culture medium consisted of Tissue Culture Medium 199 (TCM199) supplemented with ITS (10 μg/mL insulin, 5.5 μg/mL transferrin, and 5.0 ng/mL selenium), 0.23 mM sodium pyruvate, 2 mM glutamine, 2 mM hypoxanthine, 1.25 mg/mL bovine serum albumin, and different pituitary FSH (Folltropin^®^, Veterpharm, Canada) concentrations, as 10 ng/mL [as reported for another rodent, the mouse; Hardy et al. (2017)] or 50 ng/mL [as reported for goat; Magalhães et al. (2009a, b)]. Samples were then cultured at 38.5 °C and 5% CO_2_ in a humidified incubator. The culture medium was replaced every other day. After IVC for 6 days (Cecconi et al., 2004), ovarian fragments were fixed in 4% paraformaldehyde solution for 12 h and subjected to histological processing for analysis.

### Experimental design 2

In the second experiment (Figure 2), vitrified/warmed fragments were cultured in the presence of the best concentration of FSH established in the previous experiment (cryopreserved and cultured group). Non-cryopreserved (fresh control group) and cryopreserved but non-cultured (non-cultured group) tissues were used as controls. The PAFs were evaluated for survival and development using morphological and viability analysis.

#### Ovarian tissue vitrification – experiment 2

Ovaries of each animal were divided in 12 fragments, being four fragments immediately analyzed. For SSV (Praxedes et al., 2020), other fragments were individually placed in 1.8 mL plastic tubes containing vitrification solution consisted of MEM supplemented with 3.0 M ethylene glycol (EG), 10% fetal calf serum (FCS), and 0.25 M sucrose. After exposure to the vitrification solution for 5 min, the samples were dried using sterile gauze and placed on aluminum foil on a LN_2_ surface. Once vitrified, the samples were transferred (with nitrogen-cooled forceps) to cryovials for storage in LN_2_ at −196 °C. After two weeks, samples were rewarmed at 25 °C for 1 min and immersed in a water bath at 37 °C for 5 sec. The cryoprotectants were removed by three consecutive washes of 5 min in MEM supplemented with 10% FCS and decreasing sucrose concentrations (0.5, 0.25, and 0.0 M). After warming, four fragments were immediately analyzed and the others were cultured for 6 days, under the same conditions and with the best FSH concentration defined in first experiment and then evaluated.

### Histological evaluation

For morphological examination, the ovarian cortex fragments were dehydrated in increasing concentrations of ethanol, cleared in xylene, and embedded in paraffin wax. Then, the ovarian tissue samples were serially sectioned at 7 µm, and each 5^th^ section was assembled on slides and stained with hematoxylin and eosin for evaluation under a light microscope at a magnification of ×100.

When oocytes presented a regular shape with a homogeneous cytoplasm and well-organized granulosa cells, the PAFs were classified as morphologically normal; if they presented a pyknotic nucleus or ooplasm shrinkage with unorganized granulosa cells, PAFs were categorized as degenerated. Moreover, depending on their growing stage, PAFs were classified as primordial and primary; due to the low rate of secondary follicles commonly described for the species, this follicle category was not included in the analysis (Santos et al., 2018). Additionally, the proportions of healthy primordial and growing follicles were calculated before (fresh control) and after culturing to evaluate the follicular development at each treatment (Campos et al., 2021). Finally, follicle, nucleus, and oocyte diameters were measured only in healthy follicles (Matos et al., 2007).

### Viability analysis

The ovarian fragments were sliced using a scalpel blade, and placed on a stirrer with MEM for 10 min. After agitation, the solution was filtered in a 500 μm filtration screen and the suspension was centrifuged at 280×g for 10 min. The suspension (90 μL) containing individual PAFs was added to 10 μL of 0.4% trypan blue solution and subsequently incubated at 25 °C for 5 min. A total of 30 PAFs were evaluated per group under inverted microscopy (Nikon, Eclipse TS100, Tokyo, Japan). The PAFs were classified as viable when the oocyte and <10% of granulosa cells were not stained or were deemed non-viable when the oocyte and/or > 10% of granulosa cells were stained (Lucci et al., 1999).

### Statistical analysis

All statistical analyses were carried out using StatView 5.0 software (SAS Inc., Cary, NC, USA). Data were expressed as means and standard error of means (SEM). Results were analyzed by Smirnov–Kolmogorov and Bartlett tests to confirm normal distribution and homogeneity of variance, respectively. Comparisons among treatments regarding PAFs survival and morphological features were evaluated by ANOVA followed by PLSD Fisher. Values were considered statistically significant when P < 0.05.

## Results

### Effect of different FSH concentrations on fresh PAFs – experiment 1

**Figure 3:**
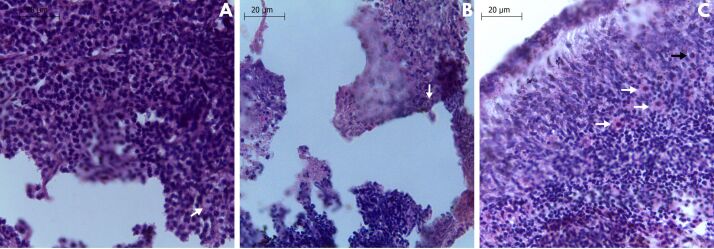
Ovarian tissue fragments derived from red-rumped agouti stained with hematoxylin-eosin. (A) Non-cultured fragments (control group) showing normal primary follicles (white arrow); (B) Cultured fragments in TCM199 supplemented with 10 ng/mL FSH for 6 days showing normal primary follicle (white arrow); (C) Cultured fragments in TCM199 supplemented with 50 ng/mL FSH for 6 days showing primordial follicles (black arrows) and primary follicles (white arrows).

Regarding PAFs morphology found (Figure 3) during the initial culture of fresh ovarian tissues from red-rumped agoutis, a total of 558 follicles was evaluated (Table 1). The positive effect of TCM199 supplemented with 50 ng/mL FSH was evident, since it provided 82.1 ± 7.4% morphologically normal follicles, a valuer significantly higher than those obtained by using 10 ng/mL FSH (61.2 ± 9.5%, P < 0.05). The effective effect of the highest FSH concentration was also verified for primordial follicles morphology (P < 0.05) (Table 1).

**Table 1:** Values (means ± SEM) for normal morphology and viability of red-rumped agouti (n = 6) preantral follicles in non-cultured group (fresh control) and in ovarian tissues cultured in TCM199 with different FSH concentrations (10 and 50 ng/mL) for 6 days.

**Treatments**	**Morphologically normal preantral follicles (%)**			**Viability** *	
	**Primordial**	**Primary**	**Total**	**%**	**Viable/Total**
Fresh control	61.5 ± 9.6^ab^ (104/129)	85.8 ± 5.50^a^ (39/50)	72.6 ± 6.7^ab^ (143/179)	90.6 ± 3.3	163/180
FSH 10 ng/mL	47.3 ± 17.0^b^ (86/117)	75.1 ± 4.7^a^ (58/66)	61.2 ± 9.5^b^ (144/183)	83.9 ± 4.8	151/180
FSH 50 ng/mL	80.1 ± 12.1^a^ (109/131)	84.1 ± 8.7^a^ (49/65)	82.1 ± 7.4^a^ (158/196)	81.7 ± 9.9	147/163

^a,b^Different superscript letters indicate significant differences in the same column (P < 0.05). *There were no significant differences among treatments regarding viability (P > 0.05).

With regards to PAFs development, there were no significant differences among experimental groups (31.5 ± 9.5%, 41.3 ± 9.9% and 54.3 ± 10.6% for fresh control, 10 ng/mL, and 50 ng/mL FSH, respectively). For viability analysis (Table 1), all the groups cultured in TCM199, in the presence of pFSH, provided values similar to those found for fresh control group (90.6 ± 3.4%). Additionally, no difference was observed between groups for follicle, oocyte, and nucleus diameters (Table 2). Therefore, considering the positive response of TCM199 supplemented with 50 ng/mL FSH for the PAF normal morphology, including primordial follicles, we considered this concentration for the second experiment.

**Table 2:** Measurements (means ± SEM) in µm of red-rumped agouti (n = 6) primordial follicles in non-cultured group (fresh control) and in ovarian tissues cultured in TCM199 with different FSH concentrations (10 and 50 ng/mL) for 6 days.

	**Fresh control**	**FSH 10 ng/mL**	**FSH 50 ng/mL**
Follicle	15.2 ± 0.6	14.8 ± 0.7	13.9 ± 0.5
Oocyte	8.9 ± 0.4	7.3 ± 0.8	7.0 ± 0.4
Nucleus	7.6 ± 0.4	7.6 ± 0.7	6.9 ± 0.7

No difference was observed between treatments (P > 0.05).

### Effect of TCM199 plus different FSH concentrations on vitrified PAFs – experiment 2

**Figure 4:**
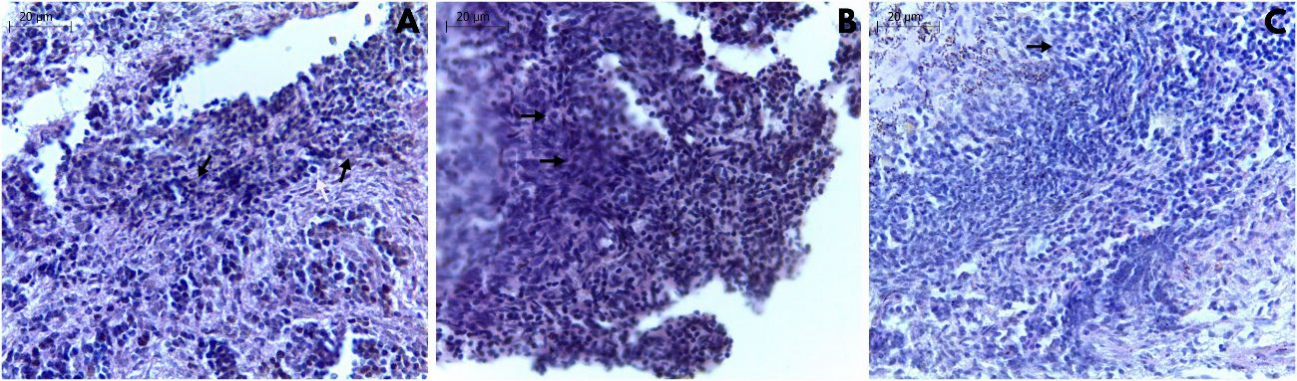
Ovarian tissue fragments derived from red-rumped agouti stained with hematoxylin-eosin. (A) Non-cultured fragments (control group) showing normal (black arrows) and degenerated (white arrow) primordial follicles; (B) Vitrified fragments by solid-surface vitrification showing normal primordial follicles (black arrow); (C) Vitrified and cultured fragments in TCM199 supplemented with 50 ng/mL for 6 days, showing primordial follicles (black arrows).

Considering the fresh and vitrified samples cultured for 6 days, a total of 299 PAFs were analyzed for morphology (Figure 4, Table 3). When compared to the control group (71.8 ± 2.1%), red-rumped agouti PAFs were efficiently preserved by SSV that presented 67.5 ± 13.9% normal PAFs immediately after warming. Moreover, a total of 76.2 ± 7.2% normal PAFs was found after 6-day IVC in TCM199 supplemented with 50 ng/mL FSH, which also presented the highest values (P < 0.05) for primordial follicles morphology (95.2 ± 4.7%).

**Table 3:** Values (means ± SEM) for normal morphology and viability of red-rumped agouti preantral follicles in fresh control group and in the samples subjected to solid-surface vitrification (SSV) and then cultured in TCM199 plus 50 ng/mL for 6 days.

**Treatments**	**Morphologically Normal Preantral Follicles (%)**			**Viability**	
	**Primordial**	**Primary**	**Total**	**%**	**Viable/Total**
Control group	68.4 ± 6.6^b^ (44/63)	73.9 ± 18.7^a^ (27/35)	71.8 ± 2.1^a^ (71/98)	88.3 ± 4.8^a^	106/120
SSV non-cultured	74.2 ± 12.4^b^ (54/79)	69.7 ± 13.5^a^ (22/40)	67.5 ± 13.9^a^ (76/119)	65.8 ± 11.4^ab^	79/120
SSV cultured	95.2 ± 4.7^a^ (24/26)	68.1 ± 9.4^a^ (37/56)	76.2 ± 7.2^a^ (61/82)	60.0 ± 9.2^b^	73/120

Different superscript letters indicate significant differences in the same column (P < 0.05).

Regarding PAFs development, we found the proportions of 73.9 ± 18.7%, 52.2 ± 13.5% and 68.0 ± 9.4% developing PAFs in the fresh, vitrified and vitrified-cultured samples, respectively. No differences were found among treatments (P > 0.05).

Immediately after warming (65.8 ± 11.4%), PAFs viability was similar to that found for fresh control group (88.3 ± 4.8%). After 6-day culture in TCM199 plus 50 ng/mL FSH, vitrified samples yet presented an amount of 60.0 ± 9.2%, similarly as those values observed immediately after warming (P > 0.05), as observed in Table 3. Additionally, no difference was observed between groups for follicle, oocyte, and nucleus diameters (Table 4).

**Table 4:** Measurements (means ± SEM) in µm of red-rumped agouti preantral follicles in fresh control group and in the samples subjected to solid-surface vitrification (SSV) and then cultured in TCM199 plus 50 ng/mL for 6 days.

	**Control group**	**SSV non-cultured**	**SSV cultured**
Follicle	14.2 ± 0.6	15.7 ± 1.1	14.5 ± 0.5
Oocyte	8.5 ± 0.7	9.47 ± 0.1	7.87 ± 0.1
Nucleus	6.8 ± 0.5	7.31 ± 1.0	5.9 ± 1.0

No difference was observed between treatments (P > 0.05).

## Discussion

To demonstrate the possibility of exploitation the female genetic material stored in biobanks, we present an initial attempt for the development of *in vitro* culture systems able to provide adequate conditions for the restoration of red-rumped agouti vitrified PAFs. Since the ovary contains thousands of follicles, collecting and preserving these follicles represent a huge opportunity for germplasm biobanking (Comizzoli et al., 2010). Therefore, the *in vitro* culture of oocytes recovered from PAFs, along with the efforts for the systematic collection and storage of germplasm, could enhance the management of endangered species populations (Campos et al., 2019).

As a media previously demonstrated for being efficient for laboratory rodents (Abedelahi et al., 2008), TCM199 also supported the fresh and vitrified PAFs culturing in red-rumped agoutis, a representative wild rodent species. This culture media is highlighted due its rich composition presenting amino acids, vitamins, ribonucleosides and deoxyribonucleosides, inorganic salts and energy sources (Mao et al., 2002). Despite its valuable compounds, however, supplements as growth factors and hormones are usually incorporated to the media to improve its effectiveness (Martins et al., 2008).

Little is known about folliculogenesis in agoutis (Santos et al., 2018), as well as which substances are involved in the initial follicular development. Based on the positive results demonstrated for domestic species (Lima-Verde et al., 2012; Saraiva et al., 2011), we choose to verify the effects of different FSH concentrations supplemented to the culture media for red-rumped agouti PAFs. In fresh samples, the most encouraging results were provided by a 50 ng/mL FSH supplementation that allowed the follicle morphology and viability preservation. These results differ from those previously other rodents, in which a 10 ng/mL FSH concentration was efficient for culturing isolate PAFs (Hardy et al., 2017), which could indicate a variate response among different species. Additionally, we highlight that the culture conditions related to the environment surrounding the follicle, which could be isolated or enclosed in the ovarian tissues, would also interfere in the efficiency of the system (Figueiredo et al., 2019).

FSH has been evidenced for promoting follicle initial growth through the stimulation of cell proliferation, steroid synthesis, and expression of receptors for epidermal growth factor (EGF) and luteinizing hormone (LH) (Martins et al., 2008). In laboratory rodents, a recombinant FSH has been commonly incorporated to the culture media for PAFs, mainly due to its high level of purity (Choi et al., 2008; Hardy et al., 2017). At the present research, however, we demonstrate the effective use of a commercial pituitary FSH for red-rumped agouti ovarian tissues IVC. Studies comparing different sources of FSH under the same conditions are still scarce. In goats, Magalhães et al. (2009a) related that rFSH was a more suitable alternative than pFSH regarding follicle culture. Ferreira et al. (2020) observed that either pFSH10 or rFSH improved the oocyte meiotic competence during the early development. If replacing pituitary with recombinant FSH could improve the results obtained for red-rumped agoutis PAFs, it remains a factor to be investigated.

As observed in previous studies (Praxedes et al., 2020), SSV provided the preservation of ~70% morphologically normal PAFs, being mostly primordial and primary PAFs. The secondary follicles are found in low quantity as described by Santos et al. (2018) for estimation of the follicular population in red-rumped agoutis. In this study (Santos et al., 2018), an average of only sixteen follicles was found per fresh ovary and one follicle was observed for vitrified ovary, which may explain the absence of follicles in the fragments submitted to culture (Praxedes et al., 2020).

Moreover, primordial PAFs submitted to the vitrification and to the culture presented greater survival than fresh control and non-cultured PAFs. The preservation of ovarian tissue is cryobiologically challenging (Santos et al., 2010) since cells at different stages can result in variations in responses to cryopreservation protocols. In mice vitrified ovarian tissues, Choi et al. (2007) observed that a major resistance of primordial PAFs to cryodamage when compared to other PAFs categories. Therefore, we can infer that the greater number of primordial normal PAFs after SSV occurred mainly because of these PAFs' resistance to extreme temperature reductions. Finally, as the primordial PAFs were better preserved than the other follicles, their proportion also increased compared to the others after SSV.

Additionally, SSV is an effective cryopreservation method that consists in an open system, being an excellent heat conductor that allows the sample to cool quickly and preserve a large percentage of morphologically normal follicles when using a lower volume of cryopreservation solution (Santos et al., 2007). Despite its efficiency, this is an open system that allows the contact of LN_2_ with the ovarian fragments, which can expose tissues to cryogenic resistant pathogens (Grout and Morris, 2009). Therefore, other vitrification systems, as the ovarian tissue cryosystem (OTC) – a closed system (Carvalho et al., 2013), have been proposed for red-rumped agouti preantral follicle preservation (Praxedes et al., 2021).

By this moment, the most effective way to provide conditions for the development of vitrified red-rumped agouti PAFs is by the xenografting to immunodeficient mice (Praxedes et al., 2018). In parallel to xenografting, the development of IVC systems is highlighted for provide essential knowledge on the folliculogenesis by evidencing the effect of individual substances on the PAFs development. Besides it, it allows to mimic the dynamics of the ovarian environment, cell communications, and interaction with secretory, hormonal, and growth factors (Figueiredo et al., 2011). At this point, the present results represent only a small step on the journey to produce an effective culture system that allow the complete development of red-rumped agouti PAFs up to ovulation and *in vitro* production of viable oocytes able to be used for other assisted reproductive technologies.

In fact, no evidence of follicle growth was observed in this study, according to morphometric analysis. Probably, other supplements need to compose the PAF culture media so that follicular activation can be observed in red-rumped agouti. This encouraging results present the perspective of improving the IVC system by trying other media as the MEM^+^ (Lima et al., 2018), other additives like the growth factors as the bone morphogenic protein 15 [BMP-15; Gomes et al. (2020)] or the growth differentiating factor-9 [GDF-9; Campos et al. (2021)], long-term culturing procedures (Choi et al., 2008), culturing isolate PAFs (Jamalzaei et al., 2016), and the use of three-dimensional systems (Asgari et al., 2015).

## Conclusion

In summary, TCM199 supplemented with 50 ng/mL FSH was efficient in maintaining the *in vitro* survival of fresh and vitrified red-rumped agouti preantral follicles. This was the first study related to the *in vitro* culture of preantral follicles included in ovarian tissue in this species, aiming to contribute to its conservation. This is a valuable information that contributes for the use of the female germplasm from wild hystricognath rodents stored in biobanks.
